# Reading with Screen Magnification: Eye Movement Analysis Using Compensated Gaze Tracks

**DOI:** 10.1145/3649902.3656493

**Published:** 2024-06-04

**Authors:** Seongsil Heo, Roberto Manduchi, Susana Chung

**Affiliations:** University of California, Santa Cruz Santa Cruz, CA, USA; University of California, Santa Cruz, Santa Cruz, CA, USA; University of California, Berkeley Berkeley, CA, USA

**Keywords:** screen magnification, eye movement, visual impairment, reading

## Abstract

Eye movements while reading with screen magnification (which requires manual scrolling to center the magnified portion of the screen within the viewport) pose interpretation challenges. Standard representations in terms of alternating fixations and saccades don’t apply to this case. This is because, during scrolling, eyes often track a moving text element, generating a movement akin to smooth pursuit. We propose a new representation that uses information from the mouse (which the reader uses to move the center of magnification) to undo the effect of magnification and scrolling. After this “compensation” operation, gaze tracks can again be described as alternating fixations and saccades. We present an analysis of gaze tracks obtained by applying this transformation on an existing dataset, recorded from low vision readers using two modalities of screen magnification. This analysis highlights similarities and differences in terms of dynamic properties of compensated gaze tracks vis-à-vis gaze during regular reading.

## 1 INTRODUCTION

For people with low visual acuity, screen magnification represents a viable computer access modality. While some individuals with low vision opt to use screen reading technology (e.g., JAWS, Apple VoiceOver, or NVDA), many prefer to read directly on the screen when possible [Szpiro et al. 2016]. In this contribution, we present an eye movement analysis for readers with low vision while using screen magnification to access onscreen content on laptop and desktop computers.

Understanding and modeling eye movements while reading proves valuable in many contexts of human-computer interaction. Algorithms for identifying intervals of “reading” vs. “skimming” on the basis of gaze time series (as measured by a gaze tracking device) were described in [Beymer and Russell 2005; Biedert et al. 2012; Campbell and Maglio 2001; Keat et al. 2003; Mozaffari et al. 2018; Strukelj and Niehorster 2018]. Being able to reliably detect when the user is reading a line of text can be useful, among other things, for automatic scrolling control. For example, in their pioneering work, Kumar et al. [Kumar et al. 2007] proposed multiple gaze-contingent modalities for vertical scrolling that adapted to the user’s reading speed. Similarly, Sharmin et al. [Sharmin et al. 2013] experimented with a system that started and stopped scrolling based on whether gaze fixation events were classified as “reading” or “not reading”.

While this prior work was concerned with vertical scrolling of *unmagnified* text, *horizontal* scrolling is paramount for reading with screen magnification. We will consider here two standard magnification modalities: *full screen* and *lens*. When using the *full screen* modality, which expands the screen content isotropically, the user scrolls the magnified content right-to-left (by moving the mouse left-to-right) to ensure that the portion of text currently read is located in their preferred region within the screen viewport [Buscher et al. 2010; Tang et al. 2023]. With the *lens* modality, only the content within a rectangular area centered around the pointer is magnified. The pointer (and thus the window of magnification) is moved left-to-right to follow the line of text while reading it. Experiments with gaze-contingent modalities for magnification control have been reported in recent work [Aguilar and Castet 2017; Manduchi and Chung 2022; Maus et al. 2020; Pölzer et al. 2018; Schwarz et al. 2020]. These systems were designed to move the center of magnification based on the location of the gaze point (point of regard on the screen), without consideration of the characteristic dynamic properties of gaze observed while reading text. Arguably, being able to determine intervals of active reading could be beneficial for magnification control, as it was proven to be for vertical scrolling of unmagnified text [Kumar et al. 2007; Sharmin et al. 2013].

The eye movement patterns during text reading are well understood [Rayner 1998], with powerful models such as EZ Reader [Reichle et al. 2003] and SWIFT [Engbert et al. 2005] enabling prediction of scanpaths (sequences of fixations and saccades). However, these models cannot be directly applied to reading with screen magnification. This is because the magnified text is continuously moved around, and thus there is not a one-to-one direct relation between the location of gaze point and the text element being gazed at. A particular text character at a certain location in a document appears on the magnified screen in a location that is a function of the location of the center of magnification (controlled by mouse or trackpad), and of the magnification factor. When one scrolls the magnified screen content with the mouse, the same text character will move on the screen. A direct consequence of this is that the eye movements during reading with screen magnification can no longer be modeled as a sequence of fixations and saccades as with normal reading. During scrolling, rather than fixating a location on the screen, readers tend to track a text element as it moves on the screen, with an eye movement that can be characterized as smooth pursuing [Wang et al. 2023].

**Figure 1:**
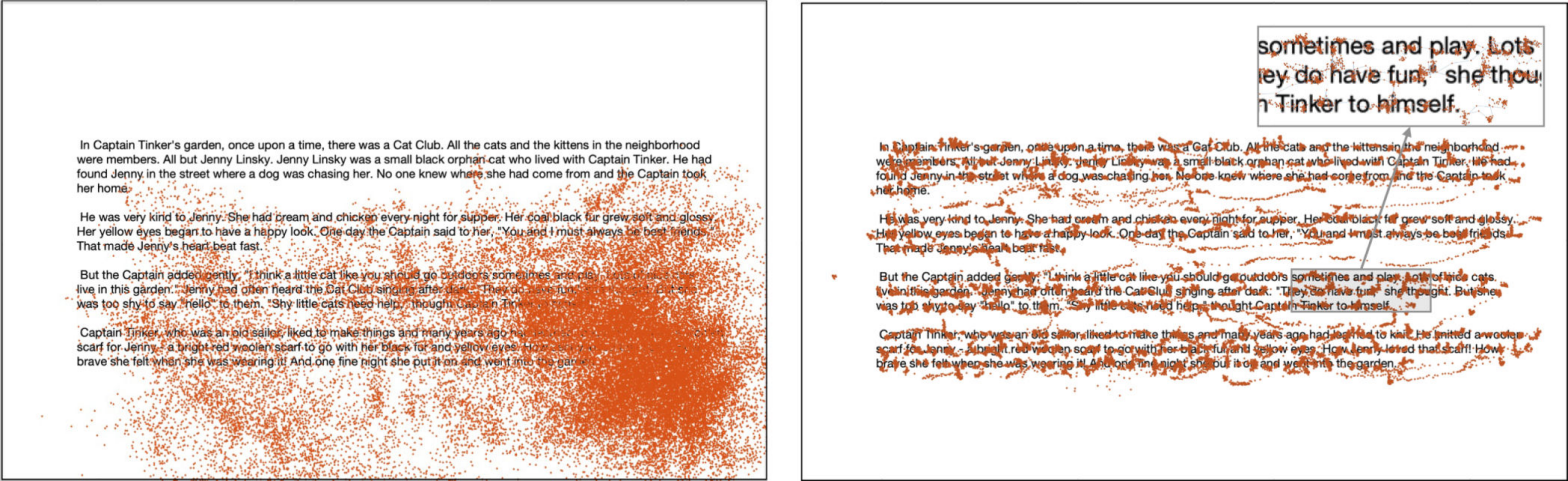
Left: Gaze points recorded from a reader with low vision using *full screen* magnification to read the text document shown (unmagnified) in the background (participant P1; magnification factor: 19.2). The reader moved the magnified screen with the mouse to bring the content of interest at each time within the screen viewport. Right: The same gaze points after compensation. Note that the compensated gaze points tend to follow the text lines. A careful observer may note clusters of compensated gaze points in the zoomed-in area, corresponding to compensated fixations.

**Figure 2:**
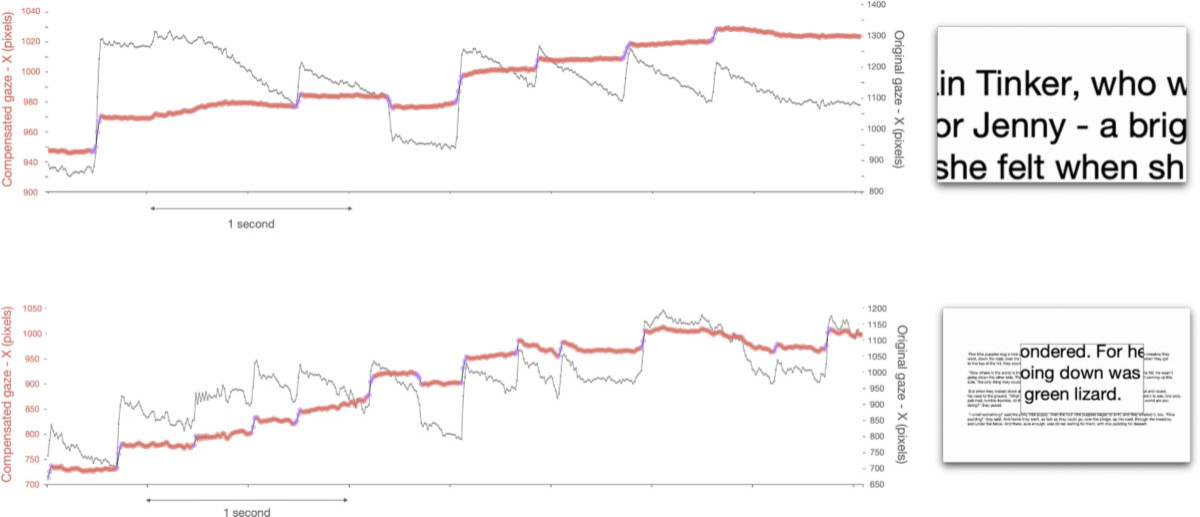
Examples of gaze tracks (X coordinate) recorded while reading magnified text. Gray: Original gaze (note the characteristic sawtooth-like profile). Red: Compensated gaze. Intervals classified as saccades are shown in purple. Top: Participant P18 using *full screen* magnification (magnification factor: 9.0). Bottom: Participant P14 using *lens* magnification (magnification factor: 4.6). For both cases, we show a sample of the magnified screen to the right.

Deciphering the gaze pattern of a reader using magnification with the purpose of associating it with the text being read can be complex (see Fig. 1, left). Fortunately, this operation can be greatly simplified if one can access the location of the pointer (and thus of the center of magnification), and use this information to “undo” the effect of magnification and scrolling. This process, which we dub *gaze track compensation*, is an original contribution of this article. Whereas gaze points, as measured by a gaze tracker, are defined in screen coordinates, a compensated gaze point identifies the current gaze location in the text document. A direct benefit of working with compensated gaze is that it simplifies gaze track analysis. The set of gaze points recorded when the reader’s gaze tracks a moving text element “coalesce”, as an effect of compensation, into relatively constant positional values (see Fig. 2). In practice, smooth pursuit intervals are transformed into *compensated fixations*, and compensated gaze tracks can again be represented as sequences of alternating compensated saccades and compensated fixations. Compensation moves gaze points close to the text lines being read, and compensated gaze tracks resemble regular gaze tracks as observed in unmagnified text reading (Fig. 1, right).

In this contribution, we present a statistical analysis of uncompensated gaze tracks, derived from original gaze data in an existing data set [Tang et al. 2023]. The data considered in this paper was recorded from 18 readers with low vision using screen magnification. We provide details of the compensation process, and present statistics of duration and extent of compensated saccades and compensated fixations, and their relation with associated eye movements.

A recent paper by Wang et al. [Wang et al. 2023] also analyzed the pattern of fixation, saccades, and smooth pursuit for readers of low vision using screen magnification. However, this previous work used raw (uncompensated) data, which means that smooth pursuit periods were considered differently from fixations. Under the compensation framework, smooth pursuits are transformed into *compensated fixations*. While Wang et al. only provided statistics of raw eye movements, our approach enables analysis of actual reading progression in reference to the text document. This allows us to directly compare the dynamic properties of compensated gaze when reading with magnification with the equivalent properties during normal reading.

## 2 PRELIMINARIES: EYE MOVEMENTS WITH AND WITHOUT SCREEN MAGNIFICATION

During reading, one’s eye movements alternates between *fixations* (periods of time during which gaze is relatively still) and *saccades*, (rapid movements during which vision is functionally suppressed). The duration of fixations is normally 200–250 ms, though its variability can be rather large (from under 100 ms to 500 ms for the same reader [Rayner 1998]). Saccades are more rapid (20–40 ms). While the vast majority of saccades (90% in skilled readers) move the gaze point forward in the text line, occasional backwards saccades (*regressions*) are observed. The average length of forward saccades is of 7–9 letter spaces (for skilled readers with normal vision); regressions are normally smaller [Rayner and Pollatsek 2006].

When the whole screen content is magnified, a mechanism must be in place to move the portion of interest of the magnified content within the physical viewport. One such mechanism is dynamic scrolling (or DRIFT) [Harland et al. 1998], which presents magnified text horizontally scrolling right-to-left at a constant speed. A more standard approach is to leave the user in charge of the motion of the center of magnification using the mouse or trackpad. In either case, gaze tracks display a characteristic sawtooth-like time profile (see [Akthar et al. 2021; Harland et al. 1998; Harvey and Walker 2014] for dynamic scrolling, and [Wang et al. 2023] for manually controlled screen magnification). Fig. 2 shows examples of this phenomenon for both *full screen* and *lens* magnification. Eye movements while reading in these conditions can thus be modeled as alternating saccades and smooth pursuit (akin to optokinetic nystagmus [Büttner and Kremmyda 2007]), during which the reader tracks a moving text element. The angular velocity of gaze in the smooth pursuit phases is constant for dynamic scrolling (as text is shown scrolling at constant speed). In the case of manually controlled magnification, the velocity (the slope of the trace seen in Fig. 2) depends on the speed of the center of magnification controlled by the mouse, and on the magnification factor. This is the reason why the gaze track slope during smooth pursuit intervals is variable, occasionally becoming flat (a *de facto* fixation) when one stops moving the mouse. An analysis of mouse movements from low vision readers using full and lens magnification was presented in [Tang et al. 2023].

## 3 METHOD

### 3.1 Dataset and Participants

We use the dataset described in [Tang et al. 2023], which was made available to us by the authors. This dataset contains gaze and mouse recordings from 30 participants with low vision, collected while they were reading text documents on the screen with both magnification modalities (for more details on the data collection, please see [Tang et al. 2023]). Text was presented on a MacBook Pro (285 × 179 mm pixel area, 2560 × 1600 pixels), and magnified using native MacOS (from the Accessibility System Settings). Gaze data was recorded by a binocular Tobii Spectrum tracker at 120 Hz. Mouse data was recorded at 10 Hz. Both data series were synchronized to a common time base.

Gaze data in this dataset was not uniformly accurate or reliable. In some cases, gaze tracker calibration (which is necessary to adapt to each users’ characteristics [Nyström et al. 2013]) was unsuccessful, making the recorded data unreliable. In other cases, there was a large proportion of undetermined gaze samples (marked as NaN in the data returned by the Tobii tracker). We culled 18 participants from the original set, based on the proportion of NaNs recorded in the files, and on the general quality of recorded gaze (discarding data from two participants because too noisy for analysis). However, for one participant, the data quality was acceptable only for *full lens* data, and thus we removed *lens* data for this participant. The proportion of undetermined data often varied between the two eyes; we decided to analyze data for the eye with the lower amount of NaNs (as reading eye movements are generally conjugate, and there are no reasons to believe that this assumption was violated in the experiment).

The participants’ age in the chosen set ranged from 32 to 95 years (mean: 70). Gender was equally represented (9 female, 9 male). The participants’ visual acuity ranged from 0.24 to 1.04 logMAR, or 20/35–2 to 20/219–2 in Snellen units (mean: 0.79 logMAR, or 20/123–2 Snellen). Participants were at liberty to choose a desired magnification factor. Chosen magnification factors ranged from 1.5 to 19.2 (mean: 5.4). Following [Tang et al. 2023], we also consider the *preferred angular print size* (PAPS) [Bailey et al. 2003]. This is the angle subtended by an x-height character at the reader’s location. PAPS is a more meaningful measure than the magnification factor as it also accounts for the viewer’s distance to the screen. PAPS values ranged from 0.64° to 1.77° (mean: 1.03°). When using the *lens* modality, participants were at freedom to choose the magnifying window width and height. Chosen window widths range from 119 to 833 pixels (mean: 582). Window heights ranged from 79 to 555s (mean: 238).

In our analysis, we only considered *in-line* data and excluded retracing periods (periods between the time users finished reading one text line and the time they started reading the next line). Retracing involves moving the center of magnification such that the beginning of the next line is within the viewport, an operation that can take substantial time (18% of the reading time [Tang et al. 2023]). Timestamps for each in-line reading period were provided with the dataset.

### 3.2 Gaze Compensation

We describe our procedure for gaze compensation here. Examples of compensated data tracks are shown in Fig. 2. Denote by $CM=\left(CM_{x},CM_{y}\right)$ the center of magnification in pixels, by MF the magnification factor, and by $N=\left(N_{x},N_{y}\right)$ the screen size in pixels. The MacOS Accessibility Systems Settings panels offer several options for *full* magnification control. Using the simplest one (“Zoomed image moves continuously with pointer”), a generic point $p=\left(p_{x},p_{y}\right)$ on the screen would be displayed after magnification, at location $p^{\left(m\right)}=CM+MF(p-CM)$. The option “Zoomed image moves to keep pointer centered”, which was used in the data collection of [Tang et al. 2023], additionally moves the location at the center of magnification CM to the center of the screen:

(1) $$p^{\left(m\right)}\to p^{\left(m\right)}+\frac{N}{2}-CM=MF(p-CM)+\frac{N}{2}$$

Hence, a point $p^{\left(m\right)}$ in the magnified screen displays the color of the original point p as per:

(2) $$p=\frac{p^{\left(m\right)}}{MF}-\frac{N}{2MF}+CM$$

The center of magnification CM is set to be equal to the location of the pointer $P=\left(P_{x},P_{y}\right)$, except when this would cause displaying points that are outside of the original screen area $\left[1,N_{x}\right]\times \left[1,N_{y}\right]$. It is easily seen that this constraint is satisfied so long as:

(3) $$\frac{N_{x}}{2MF}\leq CM_{x}<N_{x}-\frac{N_{x}}{2MF},\frac{N_{y}}{2MF}\leq CM_{y}<N_{y}-\frac{N_{y}}{2MF}$$

which leads to the following equation for CM:

$$CM_{x}=\max\left(\frac{N_{x}}{2MF},\min\left(N_{x}-\frac{N_{x}}{2MF},P_{x}\right)\right),$$

$$CM_{y}=\max\left(\frac{N_{y}}{2MF},\min\left(N_{y}-\frac{N_{y}}{2MF},P_{y}\right)\right)$$

Eqs. (2) and (4) define the mapping of the location of a screen point being gazed at, $p^{cm}$, defined in the screen coordinate frame, to the location of the same point in the reference frame of the unmagnified document, p. This represents the “compensated gaze point”.

For the *lens* magnification mode, Eq. (4) applies, with CM equal to the pointer location P. However, only the content within a window (lens) of size $W=\left(W_{x},W_{y}\right)$ is magnified. In formulas:

(4) $$p^{\left(m\right)}=\{\begin{array}{cc} CM+MF(p-CM) & \text{for}\;\left|p_{x}-CM_{x}\right|\leq \frac{W_{x}}{2MF},\;\left|p_{y}-CM_{y}\right|\leq \frac{W_{y}}{2MF}\\ p & \text{for}\;\left|p_{x}-CM_{x}\right|>\frac{W_{x}}{2},\;\left|p_{y}-CM_{y}\right|>\frac{W_{y}}{2} \end{array}$$

Note that screen points p with $W_{x}/(2MF)<\left|p_{x}-CM_{x}\right|\leq \left(W_{x}/2\right)$ or $W_{y}/(2MF)<\left|p_{y}-CM_{y}\right|\leq \left(W_{y}/2\right)$ are not reproduced in the magnified screen. This is the well-known self-occluding effect of the *lens*-type magnification [Robertson and Mackinlay 1993]. The compensation formula is thus:

(5) $$p=\{\begin{array}{cc} \frac{p^{\left(m\right)}-CM}{MF}+CM & \text{for}\;\left|p_{x}^{\left(m\right)}-CM_{x}\right|\leq \frac{W_{x}}{2},\;\left|p_{y}^{\left(m\right)}-CM_{y}\right|\leq \frac{W_{y}}{2}\\ p^{\left(m\right)} & \text{otherwise} \end{array}$$

This formula needs to be modified when the lens window reaches the edges of the screen; in these cases, the center of magnification CM continues to move with the pointer, but the window is “frozen” in location until CM is again far enough from the screen edge. We omit the related formulas here for brevity’s sake.

### 3.3 Measurements

In our analysis, we were interested in: (1) Verifying the extent to which compensated gaze tracks can be modeled as sequences of compensated fixations and saccades as in regular reading; (2) Evaluating the effect of the chosen screen magnification on eye movements; (3) Comparing the dynamic characteristics of gaze tracks recorded for *full screen* vs. *lens* magnification. For these purposes, we took the measurements as described below, involving the X coordinates of compensated and uncompensated gaze points. Note that each measurement is averaged across all data collected for each participant, resulting in one mean value per participant.

For intervals classified as compensated saccades, we computed the average duration and extent (in letter spaces) of forward and backward compensated saccades, as well as the associated angular extent of eye movement (in degrees). Saccades on compensated gaze tracks were detected using a Random Forest classifier [Birawo and Kasprowski 2022].

For intervals that are not classified as compensated saccades (i.e., for compensated fixations), we measured the duration, extent (in letter spaces), and angular extent of eye movement (in degrees). Note that, while with normal reading fixations are assumed to be relatively stable, a compensated fixation typically involves a smooth pursuit eye movement. Hence, it is reasonable to expect that even after compensation, there may be some residual dispersion of gaze (because gaze may not track the scrolling text perfectly)^1^.

We investigated the effect on these measures of the participants’ preferred angular print size (PAPS) and, for the *lens* mode, of the magnifying window’s width. These tests were conducted by measuring Pearson’s correlation coefficients. We also looked for any significant effect of magnification type. For saccades, we tested the null hypotheses that the extent, angular eye movement, and duration have similar means for forward and backward saccades. These tests were conducted using paired t-tests. Statistical significance was declared when the null hypothesis was rejected at the 5% significance level (p<0.05), with Bonferroni correction in case of multiple comparisons.

86% of all compensated saccades were found to be forward saccades. Compensated saccades alternated with compensated fixations at an average rate of 2.77 per second. The magnification modality was not found to have an effect on this data.

## 4 RESULTS

We found that the duration of fixation and saccades in our compensated data are consistent with those observed during regular reading. The average duration of fixations in our compensated data is 331 ms, falling within the 100–500 ms range typical of regular reading. Furthermore, the average duration of saccade in our compensated data is 31 ms, falling within the 20–40 ms range observed in regular reading [Rayner 1998]. As shown in Fig. 2, after compensation, interval of smooth pursuit are transformed by compensation to fixation-like intervals, where the gaze point is relatively stationary.

No significant difference in mean letter extent or mean angular eye movement was found between forward and backward-compensated saccades for either magnification modality. The magnification modality was found to have an effect on both letter extent and associated angular eye movement of compensated saccades. The mean (across participants) letter extent of compensated saccades (averaged across forward and backward saccades) was 4.3 letter spaces for *full screen* and 3.3 letter spaces for *lens*. The mean angular extent of eye movement during saccades was 3.0° for *full screen* and 2.4° for *lens*.

**Figure 3:**
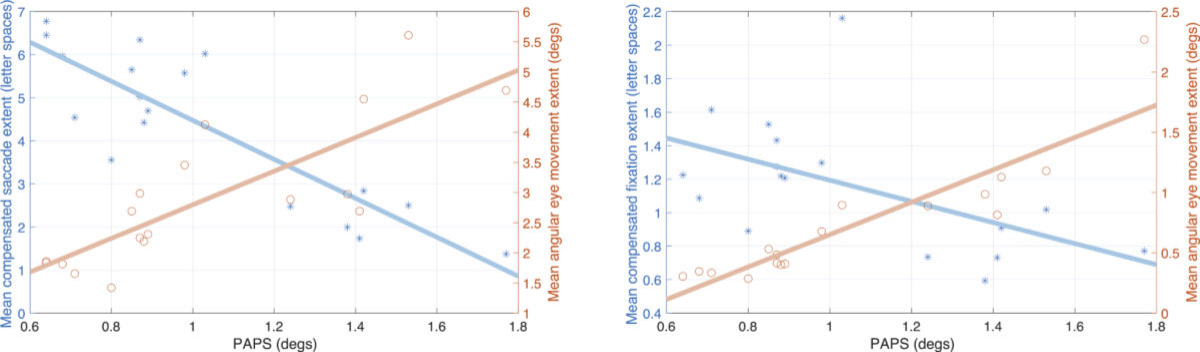
Left: The mean extent of compensated forward saccades (blue; units of letter spaces) and of the associated eye movement (red; units of degrees) as a function of the participants’ preferred angular print size (PAPS), shown together with their fitting lines (*full screen* magnification). Right: The mean extent of compensated forward fixations (blue) and of the associated eye movement(red) as a function of PAPS, shown together with their fitting lines (averaged over magnification modes).

A significant correlation was found between letter extent for compensated saccades and participants’ PAPS (ρ=−0.87 for *full screen*; ρ=−0.81 for *lens*). Likewise, a significant correlation was found between mean saccadic angular eye movement and PAPS (ρ=0.81 for *full screen*; ρ=0.75 for *lens*). Fig. 3, left, shows the letter extent and angular eye movements during saccades as functions of the participants’ PAPS for the *full screen* modality. For the *lens* mode, a positive correlation was also found between angular eye movement during compensated saccades and the width of the magnifying window (ρ=0.79).

The duration of compensated saccades was found to be significantly different between forward and backward saccades (forward: μ=36 ms; backward: μ=33ms), with no significant difference between magnification modalities. A significant correlation was found between compensated saccade duration (both forward and backward) and PAPS (forward: ρ=0.61; backward: ρ=0.78).

Compensated fixations had an average duration of 331 ms. During a compensated fixation, the compensated gaze spanned an extent of 1.16 letter spaces on average, corresponding to a mean angular eye movement of 0.73°. These values were found not to be significantly affected by magnification type. A significant correlation was found between the participants’ PAPS and both letter extent and angular motion during compensated fixations (letter extent: ρ=−0.54; angular movement: ρ=0.91). See Fig. 3.

## 5 DISCUSSION AND CONCLUSIONS

In this work, we contended that compensated gaze tracks are a convenient and effective representation of eye movements when reading with screen magnification. By using information from the mouse/trackpad (used to move the center of magnification), compensation transforms gaze tracks to become comparable to what is observed during regular reading (without screen magnification). Our analysis of experimental data highlighted both similarities and differences between the two. The duration of measured compensated fixations (331 ms on average) falls within the general range of regular fixations reported in the literature (Sec. 2), and so does the duration of saccades (33–36 ms). Forward saccades account for almost 90% of all saccades, which is also comparable with values from the literature on normal reading [Rayner and Pollatsek 2006].

Even though *full screen* and *lens* are substantially different in the way one operates the mouse to control magnification [Tang et al. 2023], recorded eye movements were found to be surprisingly similar in the two modalities after compensation. Magnification mode did have an effect on the letter extent of compensated saccades, with use of *full screen* leading to saccades that were 30% longer, on average, than for *lens*. One reason for this may be that using *lens*, due to the limited extent of the magnification window, long saccades are simply impossible, lest the gaze falls on an unmagnified portion of the screen. This may be consistent by the positive correlation found between angular eye movement during saccades and the horizontal width of the magnification window.

**Figure 4:**
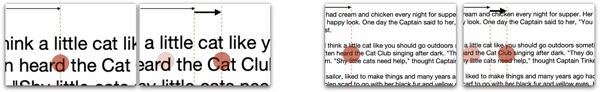
Two pairs of magnified screens (*full screen* magnification) at different magnification levels. Within each pair, the screen is shown at the beginning and at the end of a saccadic eye movement. The dark circles show the current gaze point in each frame. The dim circle in the second frame of each pair shows the location (on the document) of the previous gaze point. The thick arrow shows the actual gaze movement. Note that for smaller magnification (right), the extent of gaze movement was smaller, yet the saccade spanned a larger number of letter spaces than for the larger magnification case (left).

Our analysis found an intriguing relation between the participants’ PAPS, which is deterministically associated with the magnification factor and statistically correlated with visual acuity [Tang et al. 2023], and both letter extent and angular movement for compensated saccades (Fig. 3, left). Surprisingly, the correlation index has different signs for the two quantities. Participants with larger PAPS moved their eyes more during a saccade, and yet the number of letters spanned by this motion was smaller (see examples in Fig. 4). This may be one of the reasons why for people using screen magnification, reading speed generally decreases with increasing PAPS, as found in [Tang et al. 2023].

Another interesting observation regards compensated fixation. Our analysis found that during these periods, our participants’ gaze (while tracking scrolling text) actually spanned more than one letter space and that this amount decreased with increasing PAPS (though the associated angular span increased with PAPS, as in the case of compensated fixations; Fig. 3, right). One consequence of this is that algorithms designed to detect fixations in regular reading may need to be recalibrated to detect compensated fixations to account for a larger dispersion of gaze points.

In future work, we will explore the design of algorithms for reading/skimming discrimination while reading with screen magnification, which may enable new modalities of gaze-contingent magnification control. We will also investigate the root reasons for the poor quality of gaze data recorded for some participants, looking for explanatory factors associated with the participants’ visual conditions.

## CCS CONCEPTS

- Human-centered computing → Accessibility.

## Footnotes

1 Note that, as recommended in [Rayner and Pollatsek 2006] for regular text reading, we use (unmagnified) letter space as the unit to measure compensated saccades and fixations (where the average letter space of the unmagnified text was 2 mm, or 9.1 pixels).
